# Targeting Mitochondrial Cell Death Pathway to Overcome Drug Resistance with a Newly Developed Iron Chelate

**DOI:** 10.1371/journal.pone.0011253

**Published:** 2010-06-22

**Authors:** Avishek Ganguly, Soumya Basu, Paramita Chakraborty, Shilpak Chatterjee, Avijit Sarkar, Mitali Chatterjee, Soumitra Kumar Choudhuri

**Affiliations:** 1 Department of *In Vitro* Carcinogenesis and Cellular Chemotherapy, Chittaranjan National Cancer Institute, Kolkata, India; 2 Department of Pharmacology, Institute of Post Graduate Medical Education and Research, Kolkata, India; University of Helsinki, Finland

## Abstract

**Background:**

Multi drug resistance (MDR) or cross-resistance to multiple classes of chemotherapeutic agents is a major obstacle to successful application of chemotherapy and a basic problem in cancer biology. The multidrug resistance gene, MDR1, and its gene product P-glycoprotein (P-gp) are an important determinant of MDR. Therefore, there is an urgent need for development of novel compounds that are not substrates of P-glycoprotein and are effective against drug-resistant cancer.

**Methodology/Principal Findings:**

In this present study, we have synthesized a novel, redox active Fe (II) complex (chelate), iron N- (2-hydroxy acetophenone) glycinate (FeNG). The structure of the complex has been determined by spectroscopic means. To evaluate the cytotoxic effect of FeNG we used doxorubicin resistant and/or sensitive T lymphoblastic leukemia cells and show that FeNG kills both the cell types irrespective of their MDR phenotype. Moreover, FeNG induces apoptosis in doxorubicin resistance T lymphoblastic leukemia cell through mitochondrial pathway via generation reactive oxygen species (ROS). This is substantiated by the fact that the antioxidant N-acetyle-cysteine (NAC) could completely block ROS generation and, subsequently, abrogated FeNG induced apoptosis. Therefore, FeNG induces the doxorubicin resistant T lymphoblastic leukemia cells to undergo apoptosis and thus overcome MDR.

**Conclusion/Significance:**

Our study provides evidence that FeNG, a redox active metal chelate may be a promising new therapeutic agent against drug resistance cancers.

## Introduction

Multidrug resistance (MDR), a phenotype of cross-resistance to multiple drugs with diverse chemical structures is the major impediment of successful application of chemotherapy [1]. Although the underlying mechanisms are diverse, the role of ATP-dependent drug efflux-proteins on the cell membrane is accepted as a major cause behind MDR. Since the discovery of drug efflux proteins for last four decades, huge number of chemicals had been developed to inhibit these proteins and thus serve as resistance modifying agents (RMA). The development of non-toxic RMA capable of overcoming MDR clinically is still elusive [2], [3]. However, the basic target of chemotherapy is to induce apoptosis to cancer cells irrespective of its phenotype. Is it possible to induce apoptosis to cancer cells irrespective of its phenotype? To address this question, we had earlier showed that reactive oxygen species (ROS) plays important role in inducing apoptosis to MDR cells through generation of host protective cytokines by a copper chelate (CuNG) formed with copper salt and N-(2hydroxyacetophenone) glycinate (NHAG), synthesized by us [4], [5], [6]. In the present work we tried to understand the role of ROS generation and consequent induction of apoptosis to overcome MDR by the iron chelate (FeNG) formed with the same ligand (NHAG) [7] and iron salt. As iron is less toxic than copper and is required in high amount in normal human physiology [8], we tried to harness the ROS generating effect of ferrous ion to induce apoptosis to MDR cells.

Redox regulation has been shown to be an important component of malignant cell survival. Tipping the cellular redox balance through pharmacologic regulation in favor of increasing intracellular ROS and/or depleting protective reducing metabolites (such as glutathione (GSH) and nicotinamide adenine dinucleotide phosphate) may lead to oxidative stress and resulting in induction of apoptosis for the treatment of cancer [9]. It has been postulated that the intrinsic ROS stress associated with oncogenic transformation makes the cells highly dependent on their antioxidant systems to counteract the damaging effect of ROS and to maintain redox balance in a dynamic state (increased ROS generation and active ROS scavenging). This situation renders cancer cell highly vulnerable to further oxidative insults by exogenous agents [10]. Cancer cells, especially those in advanced disease stages, become highly adapted to intrinsic oxidative stress with up regulated antioxidant capacity. This redox adaptation generally enables the cancer cells to survive under increased ROS stress and also provides a mechanism of resistance to many anticancer agents. Owing to the presence of redox adaptation mechanisms, the use of ROS-generating agents alone may not be sufficient to kill cancer cells that have an up-regulated antioxidant capacity (mostly cellular GSH). Agents those disable such adaptive mechanisms becomes more effective against these cancer cells. Combinations of ROS-generating agents with compounds capable of abrogating cellular antioxidant systems are likely to have an additive or synergistic effect [11].

In the present work, we have synthesized and characterized a novel, nontoxic iron complex (FeNG) and harnessed its anti proliferative properties against drug resistant T lymphobastic leukemia *in vitro*. We have investigated the underlying molecular mechanisms of apoptosis upon treatment with FeNG in CEM/ADR5000 cells. The pro-apoptotic activity of iron chelate involves mitochondrial apoptotic pathway through generation of ROS and hints at a possibility of utilizing redox active metal chelates in combating cancer.

## Results

### UV_Vis spectral study

UV spectrum for the ligand λ max (water) was observed at: 211, 253 and 325 nm.

UV-VIS spectrum for the complex λ max (water) was observed at: 218, 254, 340 and 475 nm.

The electronic absorption spectrum of the complex shows four bands in aqueous solution at 218 nm, 254 nm, 340 nm and 475 nm. The first three absorption bands are also observed in almost the same λ value in free ligand. These bands are perhaps due to intra-ligand transition.

In the metal complex these three bands appear with higher λ values, as there may decrease electron donating ability after complexation of ligand. The 475 nm band in the complex may be assigned as d-d transition band, due to the spin forbidden transitions from ^6^A_1g_ to ^4^T_1g_ and from ^6^A_1g_ to ^4^T_2g_ [4]. This band position of the complex suggests an octahedral geometry of the Fe(II) complex.

### Infra Red spectral study

Important infrared (i.r.) bands for the ligand appear at: 3410−3360, 1689, 1619, 1524, 1466, 1421, 1395, 1318, 1269, 1205, 1163, 969, 931, 752 and 730 cm^−1^ [7].

Important i.r. bands for the complex appear at: 3219–3385, 1598, 1538, 1434, 1390, 1331, 1309, 1231, 1162, 1134, 1083, 1024, 963, 865, 745, 605, 525, and 433 cm^−1^.

The νCN characteristic stretching band in the ligand appears at 1619 cm^−1^ and shifts in the lower frequency region in the complex at 1598 cm^−1^; such shifting towards lower frequency region suggests the coordination between nitrogen atom of the ligand and the Fe-metal. In the ligand, one strong band appears at 1689 cm^−1^ due to asymmetric stretching vibration of –COO^–^ and another strong band appears at 1395 cm^−1^ due to symmetric stretching vibration of – COO^–^ group [7]. In the metal ligand system, 1689 cm^−1^ band is not observed and the band at 1395 cm^−1^ band shifted to 1434 cm^−1^; so there is strong indication that the COO^—^group coordinates through deprotonation. The νC-O ligand band (1269 cm^−1^) shifted towards higher frequency side in the complex at 1309 cm^−1^. This high frequency shift of νC-O phenolic band confirms the formation of covalent bond between oxygen atom of phenolic –OH and metal ion through deprotonation [12]. The –OH group participation in coordination is also indicated by the shift of 3394 cm^−1^ band (−OH group) towards 3219–3385 cm^−1^ through deprotonation and formation of a metal-oxygen bond.

Metal ligand vibrations are generally located in the region 600–250 cm^−1^. The skeletal vibrations of the ligand appearing in this region complicate the scope of interpretation. However, the comparison of the complex and ligand spectra allowed the assignment of metal sensitive bands. In the present case, we have assigned the band at 605 cm^−1^ to νM-O in the complex [4].

### Proton NMR Study

Proton nmr peak of the ligand in D_2_O appears at δ 7.38–7.51 (S, 5H) and δ 6.59–6.76 (S, 3H) for aromatic protons. -CH_2_ protons appear at δ 4.09 (1H, M). -CH_3_ protons appear at δ 2.26–2.29 (4H, M).

Proton nmr peak of the complex FeNG in D_2_O appear at δ 6.88–7.8 (5H, S) for aromatic protons. -CH_2_- protons appear at δ 3.49. CH_3_ protons appear at δ 2.55.

The characteristic proton signals due to aryl group in the ligand (δ 6.59–7.5) are almost unaffected in the complex and appear at δ 6.88–7.8. Complexation causes drastic changes of proton signals of -CH_2_ and -CH_3_ groups in the ligand. The signal in the ligand due to -CH_2_ group (δ 4.09) shifts to higher field δ 3.49 in the complex; This is an indication of considerable drift of electrons from two neighboring groups viz., −N = C and –COOH to the metal moiety. The signal for CH_3_ shifts to lower field in the complex (in the complex at δ 2.55 and in the ligand at δ 2.26–2.29) due to deshielding of protons and indicates participation of CH_3_-C = N- group to coordination with iron atom [4].

### Mass Spectral Study

**Figure 1:**
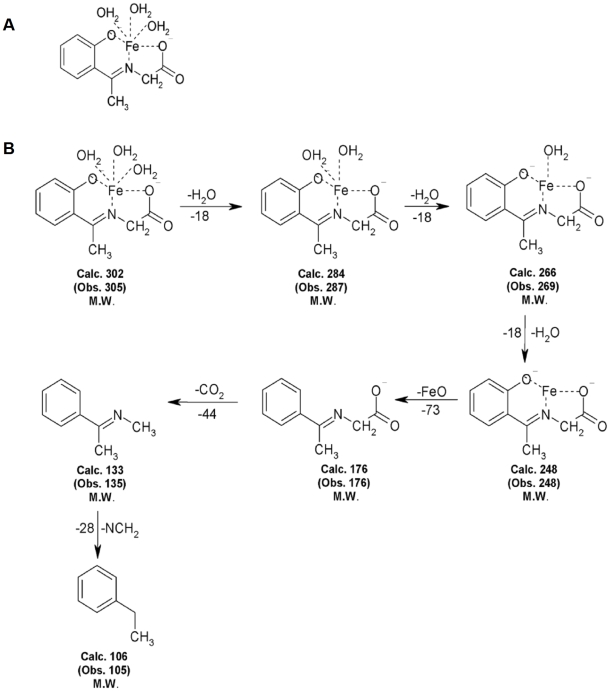
Structure and Mass spectral study of Iron Complex. (A)Chemical Structure of iron complex, iron (II) N-(2-hydroxyacetophenone) glycinate (FeNG). (B) Mass fragments of FeNG.

The formation of molecular ion peaks indicates that the structure of the iron complex in Fig. 1A and Mass spectral data is presented in Fig. 1B.

### Antiproliferative effects of FeNG

**Figure 2:**
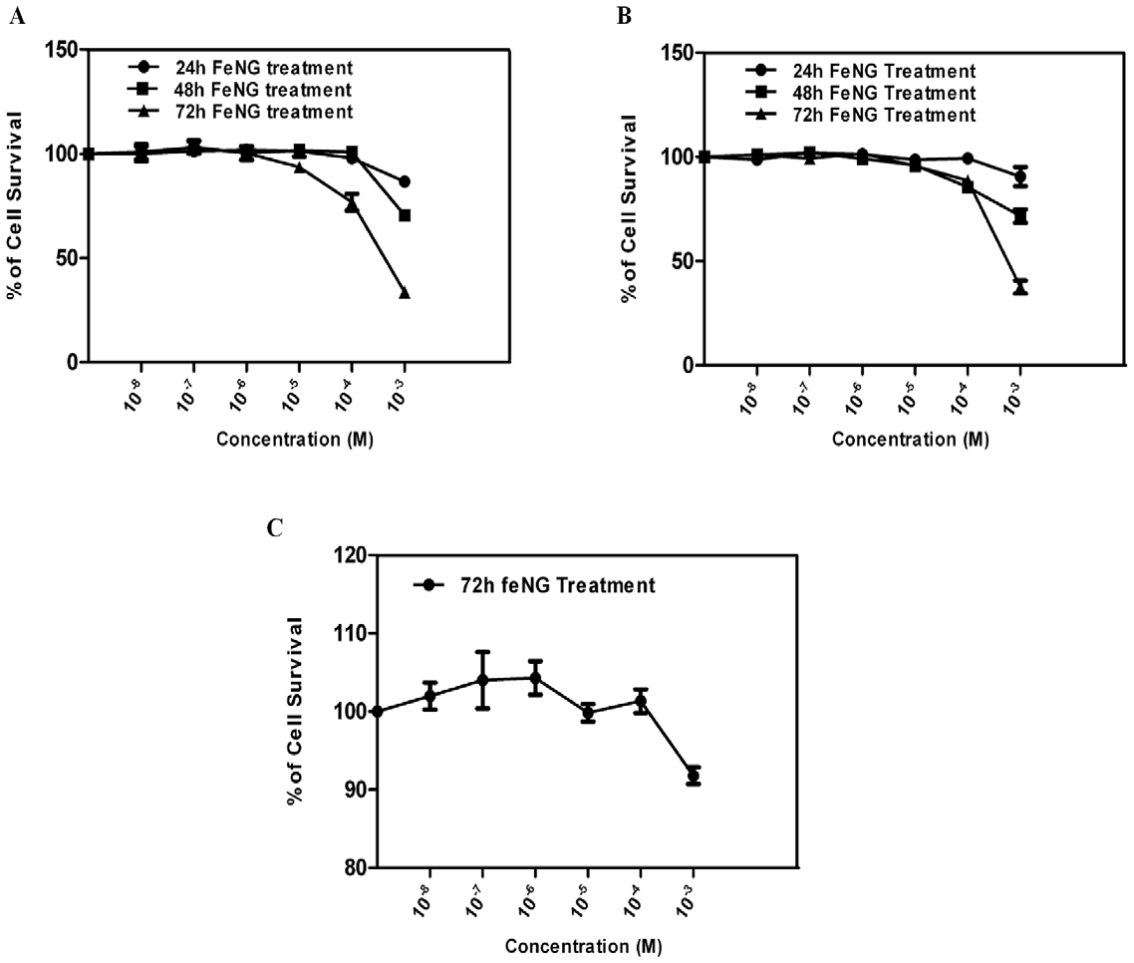
Comparison of the cytotoxic effect of iron complex on different cell types. Dose response curves for iron complex (FeNG) using (A) CEM/ADR5000 (B) CCRF-CEM, and (C) Human PBMC cells, as assessed by MTT assay. Cells were seeded into 96-well plates (4×10^4^ cells/well) and allowed to overnight incubation at 37°C in 5% CO_2_ incubator. Next day, cells were treated with increasing concentrations of FeNG for 24 h, 48 h, and 72 h incubation. Results are expressed as percentage viability of solvent-treated control cells. Value represents the mean ± SD of three independent experiments with four replicates in each.

In an initial approach, to determine the antiproliferative effect of FeNG on T lymphoblastic leukemia cells we performed MTT (3-[4, 5-dimethylthiazol- 2-yl]-2, 5-diphenyltetrazolium bromide) assay employing CEM/ADR5000 in comparison to CCRF-CEM or human PBMC (peripheral blood mononuclear cells). FeNG induced growth inhibitory effect occurred in time as well as dose dependent manner in CEM/ADR5000 (fig. 2A) and CCRF-CEM (fig. 2B) cell line with IC_50_ values (at 72 h treatment) 0.75×10^−3^ M and 0.79×10^−3^ M respectively (Table 1). However under the same condition FeNG didn't display cytotoxic effect on normal human PBMC (fig. 2C) at given experimental concentration range. The results presented in the Table 1 would suggest that CEM/ADR5000 and CCRF-CEM cells were equally sensitive to FeNG as the difference in IC_50_ values between two different cell lines were statistically not significant. In addition, data obtained for FeNG displayed a considerable lower resistance factor than doxorubicin [13] suggested that the complex was not a potential MDR1 substrate (Table 2).

**Table 1:** IC_50_ Values of FeNG for CEM/ADR5000, CCRF-CEM and Human PBMC.

	IC_50_ values (mM±SD)		
Compound	CEM/ADR5000	CCRF-CEM	Human PBMC
FeNG	0.75±0.06	0.79±0.11	Not determined

Anti-proliferative activity of FeNG was determined using CEM/ADR5000, CCRF-CEM, and Human PBMC following 72 h continuous incubation. All the data are representative of three similar experiments. Values represent mean ± S.D.

**Table 2:** Calculation of Resistance factor for FeNG.

	IC_50_ values (mM±SD)		
Compound	CEM/ADR5000	CCRF-CEM	Resistance factor
FeNG	0.75±0.06	0.79±0.11	0.95
Doxorubicin	0.00025±0.0001	0.1±0.009	400*

Anti-proliferative activity and resistance factor used to confirm multi-drug resistance phenotype and demonstrating whether iron complex (FeNG) was a substrate for P-glycoprotein. The resistance factor was calculated by division of the IC_50_ for the drug resistance CEM-ADR 5000 cell line by the IC_50_ for the drug sensitive CCRF-CEM cell line. Results presented are representative of three independent experiments.

*Reference[13].

### Selective cellular and nuclear morphological changes in CEM-ADR5000 cells after FeNG treatment

**Figure 3:**
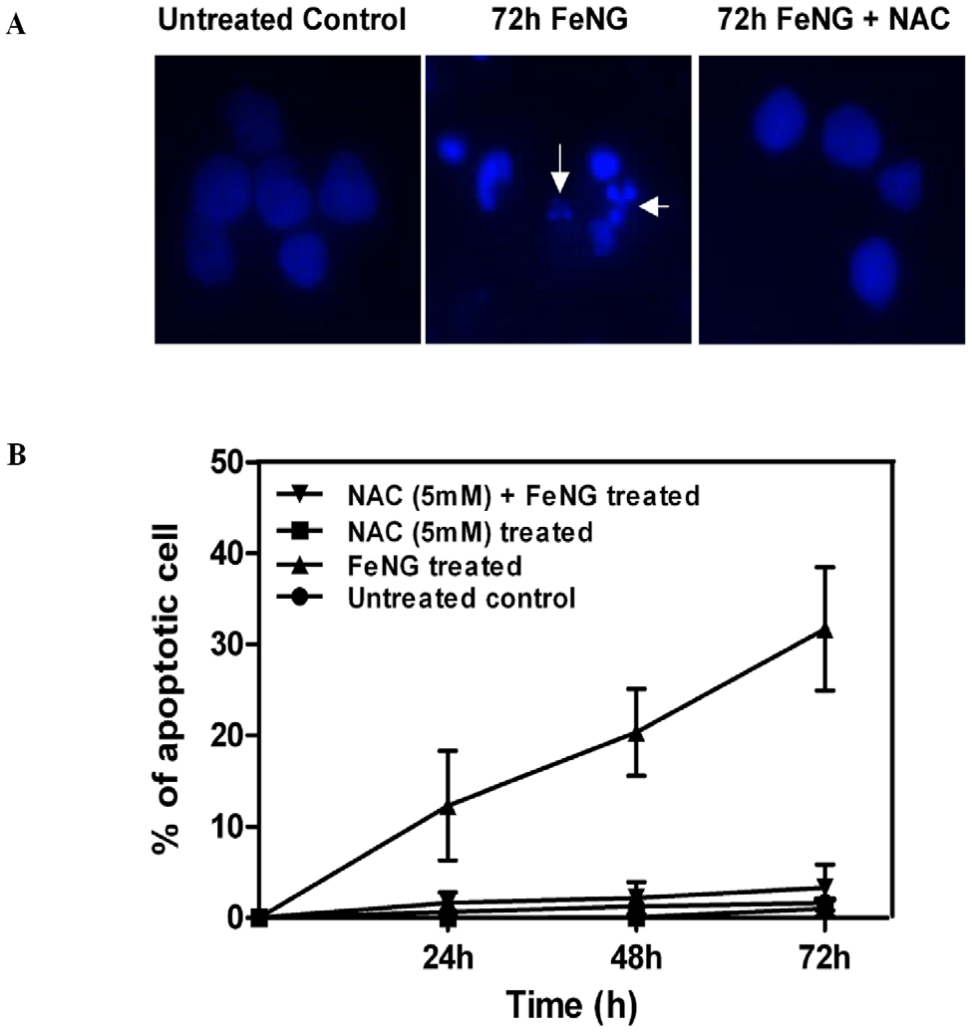
Changes in nuclear morphology of CEM/ADR5000 cells after FeNG treatment. (A) Morphological changes of CEM/ADR5000 cells treated with 0.75×10^−3^ M FeNG alone or in combination with 5 mM NAC (one hour prior to FeNG treatment). CEM/ADR5000 cells after treatments with drugs were fixed with 1% paraformaldehyde and stained with Hoechst 33258. The cells were observed under a fluorescence microscope. Apoptotic cells showed condensed or fragmented chromatin in the nucleus (arrowhead). (B) Represents the temporal kinetics of apoptotic percentage of CEM/ADR5000 cells. Cells were treated with FeNG alone or in combination with 5 mM NAC for the indicated times. After treatment, cells were harvested and stained with Hoechst 33258. Apoptotic cells were examined by counting the cells with condensed and fragmented nuclei. Each point represents an average of three independent experiments, and standard deviation bars are shown.

The Hoechst 33342 staining is sensitive to DNA and was used to assess changes in nuclear morphology. A concentration of 0.75×10^−3^ M is high enough to inhibit cell growth (fig. 2A). CEM/ADR5000 cell were treated with FeNG for 24 h, 48 h, and 72 h and the iron complex induced nuclear condensation as well as nuclear fragmentation (a typical apoptosis associated markers) was determined by fluorescence microscopy (fig. 3A). As shown in fig. 3B, percentage of apoptotic cells was increased in time dependent manner when cells were exposed to FeNG.

### FeNG induces apoptosis in CEM/ADR5000 cell lines in time dependent fashion

**Figure 4:**
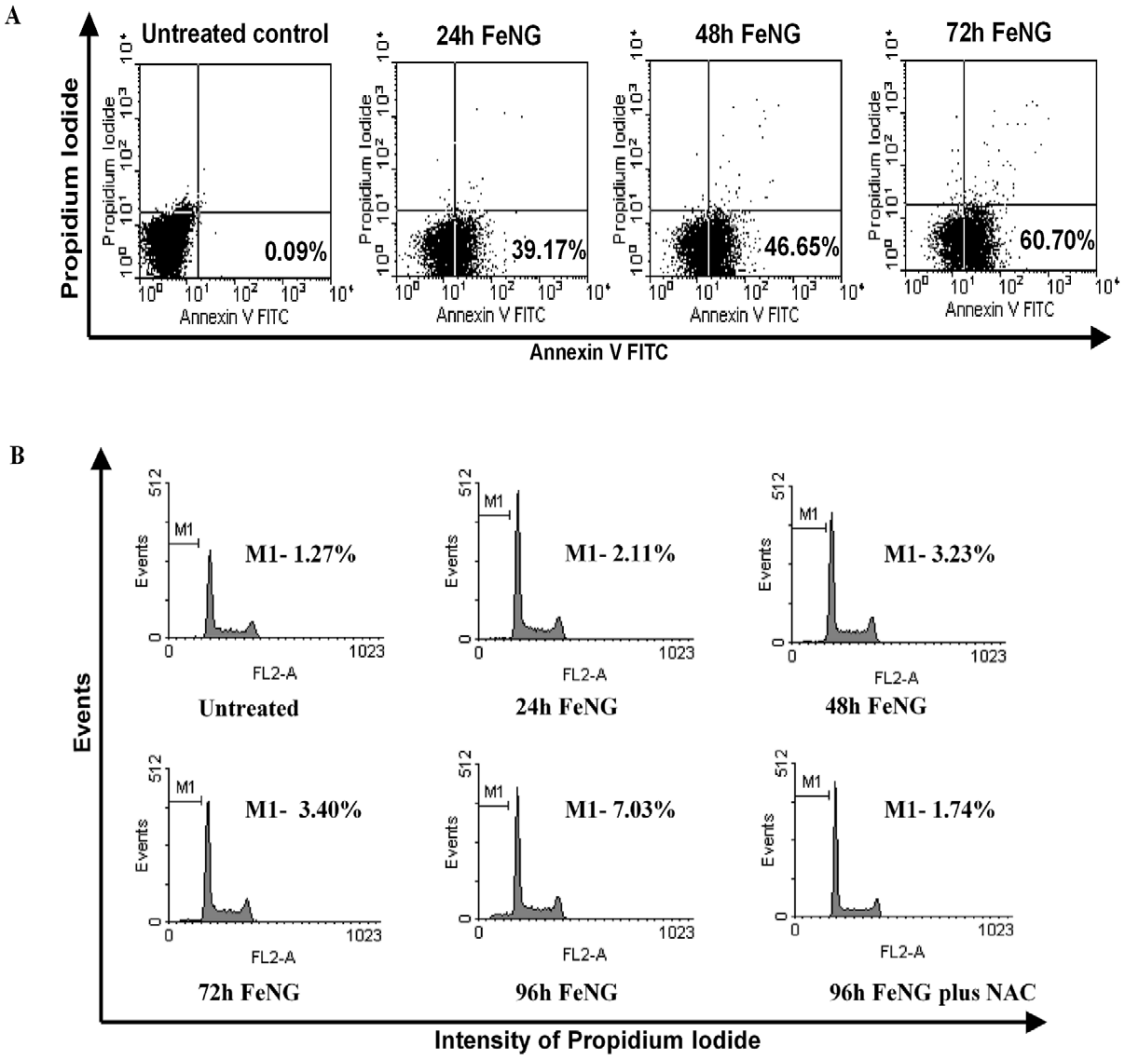
FeNG induces apoptosis in CEM/ADR5000 cell line. (A)CEM/ADR5000 cells were incubated with FeNG for the indicated time and then stained with annexin- FITC, which specifically detects exposed phosphatidyl serine residues at the cell surface. The number of annexin-V-positive cells was determined using a flow cytometer and the percentage is represented in each panel. (B) Cell cycle distribution of CEM/ADR5000 cells after FeNG treatement. CEM/ADR5000 cells treated with FeNG alone or in combination with 5 mM NAC for indicated times were harvested and fixed in 70% ethanol. After staining with propidium iodide they were analyzed using a flow cytometer. The percentage of cells in the sub-G1 (representative of hypodiploid DNA content) population is indicated in each panel.

Early cellular changes in apoptosis are characterized by the translocation of phosphatidylserine (PS) to the external surface of the plasma membrane where it can be detected by binding to annnexin V- FITC. As cell membrane is further compromised and cell death occurs, cellular DNA becomes accessible for staining with PI [14], [15]. The flow cytometric analysis showed that (fig. 4A), the CEM/ADR5000 cells that had been incubated with FeNG for 24 h, 48 h, and 72 h and dual stained with annexin V-FITC and PI, there was a progressive increase in the annnexin V-FITC positive population of cells (39.17%, 46.65%, 60.70% for 24 h, 48 h, 72 h respectively) in a temporal manner as compared to untreated control (0.09%).

In addition, FeNG induced apoptosis was also determined by cell cycle analysis of PI stained CEM/ADR5000 cell line by flowcytometry after FeNG treatment. It was found that (fig. 4B), the increase in the counts of sub diploidal (sub G1/G0) cells in a time dependent way as compared to untreated control.

### FeNG induced apoptosis involves mitochondria mediated pathway in CEM/ADR5000 cell line

**Figure 5:**
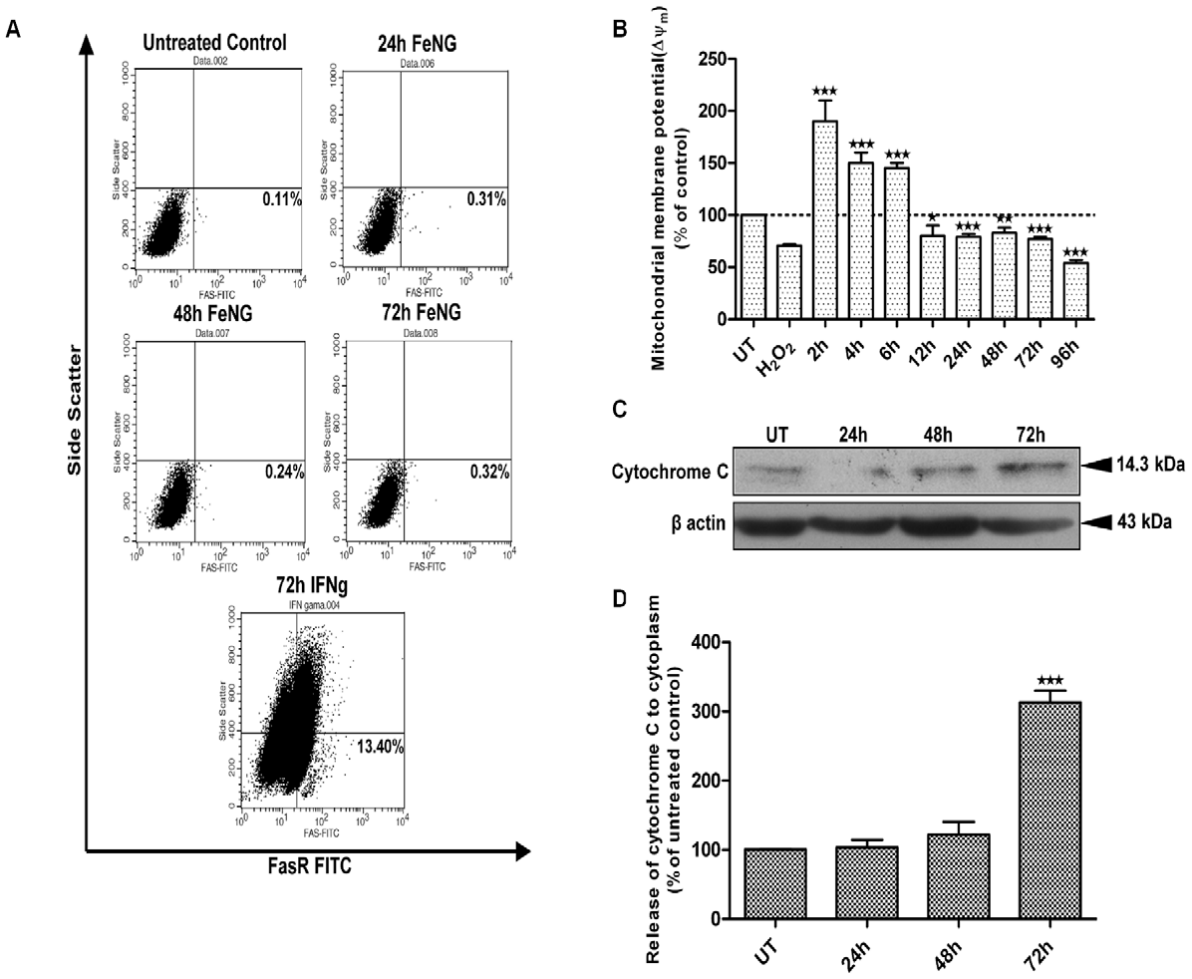
FeNG induces apoptosis through mitochondrial cell death pathway. (A) CEM/ADR5000 cells of both untreated and FeNG treated for indicated time or rIFN γ treated were labeled with anti FasR antibody. Immunofluorescence analysis was performed by flow cytometry. Representative data of 3 independent experiments is presented. (B) CEM/ADR5000 cells were treated with or without FeNG for indicated time, and mitochondrial membrane potential was measured after JC1 staining. The ratio of red fluorescence (mitochondrial JC-1) to green fluorescence (cytoplasmic JC-1) was used as a surrogate for mitochondrial potential. Data represent mean ± SD of three independent experiments. Statistically significant difference from untreated control at *P<0.05, **P<0.01, ***P<0.001, respectively. (C) Effect of FeNG on the release of cytochrome c. Western blot analysis of cytosolic extracts from CEM/ADR5000 cells treated with FeNG (10^−4^ M) for indicated hours. Cytosolic fraction was prepared as described in Materials and Methods. Membrane was probed with anticytochrome *c* antibody followed by incubation with peroxidase-conjugated secondary antibody. The protein was visualized by Lumi glow detection system. Membrane was blotted for β-actin (bottom panel) for loading correction. (D) Densitometric quantitation of cytochrome c levels in the cytoplasm. Immunoreactive bands were quantitated and expressed as the ratio of each band density to corresponding loading control (β actin) band density and values were represented after normalization to untreated control.

Since apoptotic cell death may be actuated through the extrinsic (transmembrane death receptor mediated) or the intrinsic (mitochondria mediated) pathway, we enquired which pathway was involved in FeNG induced cell death. To look into this question, we treated CEM/ADR5000 cells with FeNG for 24 h, 48 h, and 72 h and FasR expression on cell were ascertained by flowcytometry. Fig. 5A showed that FeNG was not able to induce FasR expression on CEM/ADR5000 cells. On the contrary increased expression of FasR was detected on CEM/ADR5000 cells after 72 h treatment of 100 pg/ml of human recombinant IFN-γ. This data indicated that the extrinsic pathways might not be involved in FeNG mediated apoptosis.

Cell death through the mitochondrion involves an increase in mitochondrial permeability transition that results in the release of cytochrome *c* and downstream activation of effector caspases. The increase in mitochondrial permeability transition is accompanied by a collapse in mitochondrial membrane potential (ΔΨ_m_) [16], [17] that can be measured by JC-1 dye staining. In healthy nonapoptotic cells, JC-1 is accumulated in mitochondria in proportion to inner membrane potential and form a “J aggregates” that fluoresce red; however, with the loss of mitochondrial membrane potential, the dye remains in the cytoplasm where JC-1 exist as monomer that fluoresce green. The ratio of red to green fluorescence provides a measure of ΔΨ_m_. After exposure to FeNG for 2 h to 6 h there was a time dependent increase in ΔΨ_m_, indicating that mitochondria were hyperpolarized followed by a decline in ΔΨ_m_ which was detected at around 12 h (fig. 5B). Our data indicating that exposure to FeNG results in a biphasic change in ΔΨ_m_ with an early hyper polarization, followed by a later depolarization and ΔΨ_m_ collapse.

Mitochondrial swelling induced by permeability transition is known to cause the outer membrane rupture and followed by release of cytochrome c from mitochondria to cytosol [18]. To analyse the involvement of mitochondria in the apoptosis induced by FeNG, a cytochrome c release assay was performed. As illustrated in the fig. 5C, FeNG treatment induced the release of cytochrome c to cytosol as detected by western blot analysis of cytosolic fraction. The intensity of immunoreactive band was found to increase in a time dependent fashion after FeNG treatment (fig. 5D).

### Reactive oxygen species is critical for FeNG induced apoptosis in CEM/ADR5000 cells

**Figure 6:**
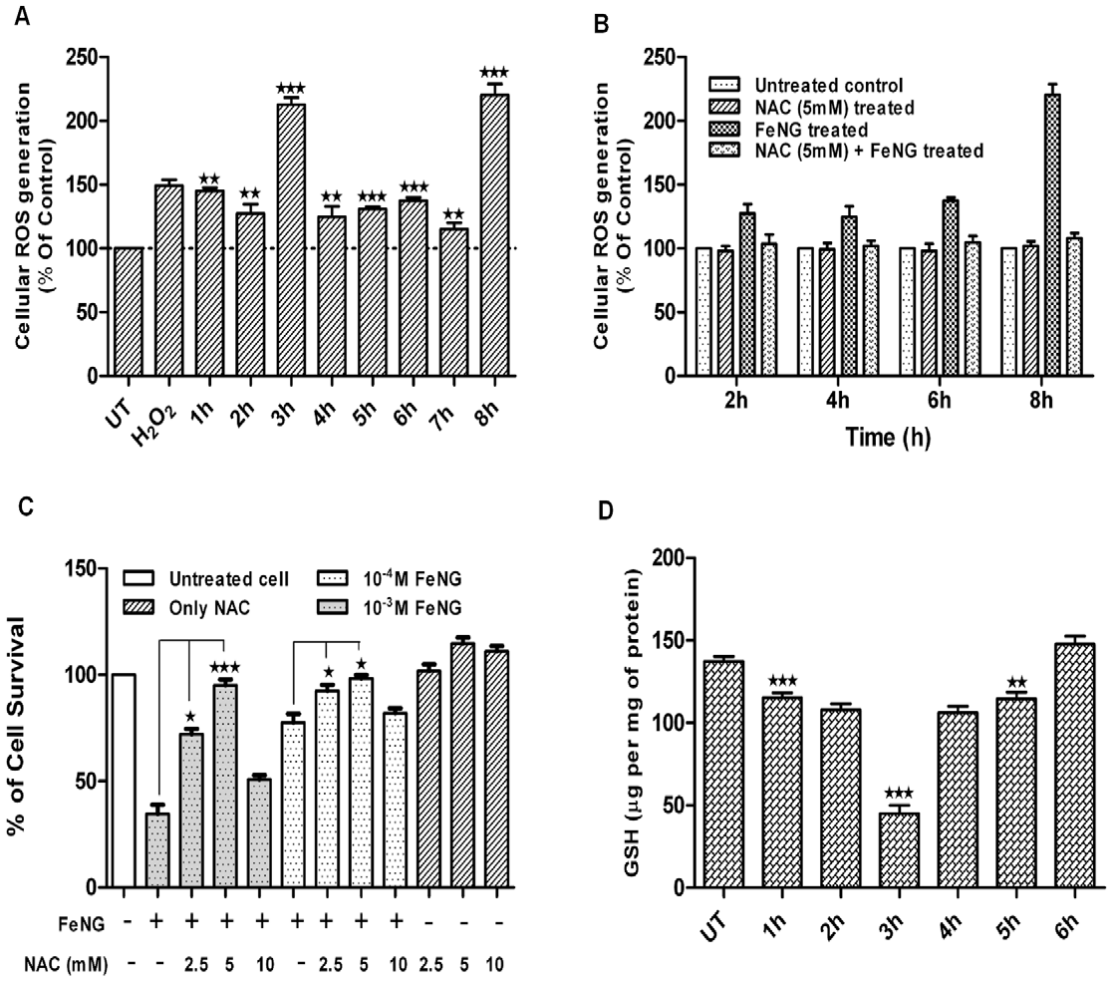
Reactive oxygen species (ROS) plays an important role in FeNG induced apoptosis. (A) CEM/ADR5000 cells were either kept untreated or treated with FeNG (10^−4^ M) and intra cellular ROS generation was measured [in terms of peroxide using dichlorofluorescein diacetate (DCF-DA)] as described under *Materials and Methods* at different time points. Data are expressed as percent of control and are presented as mean±SD of 3 independent experiments. Differences between control and FeNG treated cells are significant *P<0.05, **P<0.01, ***P<0.001, by unpaired Student's *t* test. (B) NAC completely abrogated FeNG induced ROS generation in CEM/ADR5000 cells. Cells were either kept untreated or pretreated with NAC (5 mM) for 1 h. Then the cells were further cultured for 2 h, 4 h, 6 h and 8 h in the presence or absence of FeNG (10^−4^ M) and intra cellular ROS generation was measured. (C) Represents that NAC protects CEM/ADR5000 cells from FeNG induced cell death. CEM/ADR5000 cells were either left untreated or pretreated with different concentration of NAC for 1 h. The cells were then treated with FeNG (10^−3^ M or10^−4^ M) for 72 h and cell death was monitored by MTT assay. Value represents the mean ± SD of three independent experiments with four replicates in each. Significant difference at *P<0.05, ***P<0.001, respectively, from only FeNG treated cells. (D) FeNG depletes intra cellular glutathion (GSH) contents of CEM/ADR5000 cells. Cells were either kept untreated or treated with FeNG (10^−4^ M) for indicated time points and intra cellular GSH was measured as described under *Materials and Methods*. Results are presented as mean±SD of 3 independent experiments. Differences between untreated control and FeNG treated cells are significant **P<0.01, ***P<0.001, by unpaired Student's *t* test.

The intrinsic pathway of apoptosis can be triggered by many stimuli including ROS. Mitochondria are the major site for ROS production, and accumulation of ROS may lead to the initiation of apoptosis [19]. Previously we have shown that CuNG produces ROS in EAC/DOX cell line [20], therefore in the present work we have investigated whether FeNG, structurally similar to CuNG can kill tumor cells through induction of ROS generation. We measured intracellular H_2_O_2_ using oxidation sensitive fluorescence dye DCFDA in CEM/ADR5000 cells. It was found that FeNG induced a rapid accumulation of H_2_O_2_ in CEM/ADR5000 cells and maintained a sustained elevated level of H_2_O_2_ as compared to untreated control (fig. 6A). This elevated level of ROS was completely blocked by NAC (N acetyl cystein) (fig. 6B). NAC is an aminothiol and synthetic precursor of intracellular cystein and GSH and also known as a general antioxidant, which scavenge the ROS (fig. 6B).

To further investigate whether FeNG induced ROS is required for induction of apoptosis, CEM/ADR5000 cells were treated with different concentrations of NAC 1 h prior to FeNG treatment and cell death and apoptosis was monitored by MTT assay and cell cycle analysis. The experiment showed that 5 mM NAC completely protect CEM/ADR5000 cell from FeNG induced apoptosis (fig. 6C and fig. 4B).

### FeNG depletes intracellular GSH level

Cellular redox homeostasis is maintained by the balance between ROS generation and successful elimination of ROS by cellular antioxidant capacity. Exogenous agents that increase ROS generation or decrease antioxidant capacity will shift the redox balance and result in an overall increase in the level of ROS, which when above a cellular tolerability threshold may induce cell death [11]. However, depletion of GSH levels, a hallmark of oxidative stress can be an early event that may contributes to the induction of apoptosis [21].

To assess the effects of FeNG on intracellular GSH level, CEM/ADR5000 cells were treated with FeNG for different hour and GSH was measured by fluorimetric method. It was found that GSH was depleted gradually after FeNG treatment up to 4 h and then it reaches its normal levels as compared to untreated control (fig. 6D).

### Activation of caspase 3 occurred in FeNG induced apoptosis in CEM/ADR5000 cell line

**Figure 7:**
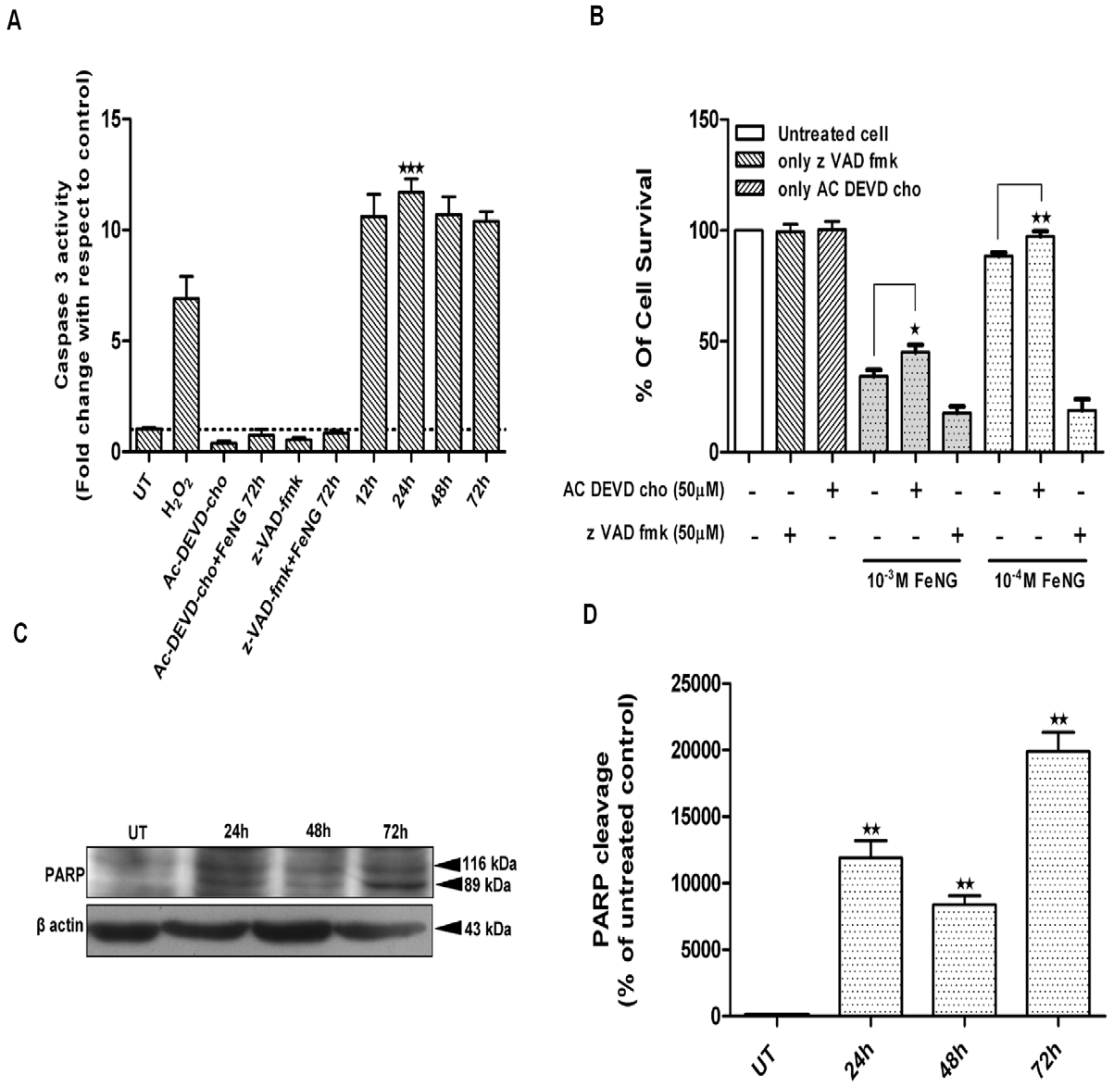
Activation of caspase 3 in CEM/ADR5000 cells after FeNG treatment. (A) Effect of FeNG and caspase inhibitors on the activity of caspase-3 of CEM/ADR5000 cells. Cells were treated with either vehicle (medium) control or FeNG (10^−4^ M) for 12 h, 24 h, 48 h, 72 h or caspases inhibitors; Ac-DEVD-cho (caspase 3 specific inhibitor) and z-VAD-fmk (pan caspase inhibitor) alone (50 µM) or in combination with FeNG for 72 h. After completion of these treatments, cells were harvested and cell lysates were prepared. The enzymatic activity of cell lysates towards tetrapeptide chromogenic substrates Ac-DEVD-AMC was determined. Caspase activities are expressed as fold change of control and presented as mean±SD of three independent experiments. Differences between untreated control and FeNG treated cells are significant ***P<0.001, by unpaired Student's t test. (B) Effects of caspase inhibitor on FeNG induced cell death of CEM/ADR5000 cells. Cells were either left untreated or treated with FeNG (10^−3^ M or10^−4^ M) or Ac-DEVD-cho (50 µM) and z-VAD-fmk (50 µM) alone or in combination with FeNG for 72 h and cell death was monitored by MTT assay. Value represents the mean ± SD of three independent experiments with four replicates in each. Significant difference at *P<0.05, **P<0.01, respectively, from only FeNG treated cells. (C) Effect of FeNG on the cleavage patterns of PARP in CEM/ADR5000 cells. Cells were grown at standard culture conditions as mentioned in Materials and methods, and treated with FeNG (10^−4^ M) for 24 h to 72 h, and cell lysates were prepared. Immunoblot analysis was performed to identify the full (116 kDa) and cleaved (89 kDa) PARP using specific primary antibodies. Loading was checked by immunoblotting of β -actin. Bands were visualized by Lumi glow detection system. Data shown are representative of three independent experiments. (D) Densitometric quantitation of cleaved (89 kDa) frgments of PARP in the cytoplasm. Immunoreactive bands were quantitated and expressed as the ratio of each band density to corresponding loading control (β actin) band density and values were represented after normalization to untreated control.

Caspases are crucial mediator of programmed cell death (PCD). Caspase 3 is a frequently activated death proteases, and required for some typical hallmarks of apoptosis and is indispensible for apoptotic chromatin condensation and DNA fragmentation in all cell types examined [22]. So in order to detect the enzymatic activity of caspase 3 during the induction of cell death by FeNG, we used a fluoregenic peptide substrate (Ac-DEVD-AMC) specific for caspase 3. Caspase activity was monitored following treatment of CEM/ADR5000 with FeNG for various intervals. As shown in the fig. 7A, the FeNG led to an increase in caspase 3 activity in CEM/ADR5000 with the onset at 12 h and reaching a maximum at 24 h which persisted until 72 h after treatment.

To determine whether the activity of caspase 3 is essential for FeNG induced cell death, the effect of a caspase 3 specific inhibitor (Ac-DEVD-cho) and a caspase family inhibitor (Z-VAD-fmk) was also investigated. As shown in the fig. 7B, caspase 3 specific inhibitor significantly but partially abrogated FeNG induced cell death in CEM/ADR5000 cell line. This result confirms that FeNG exerts apoptogenic activity through activation of caspase 3 and also indicate that involvement of other pathway(s) in FeNG induced apoptosis. Surprisingly, Z-VAD-fmk didn't able to block FeNG induced cell death, rather it enhanced cell death potential of FeNG in CEM/ADR5000 cells. This observation may be due to existence of alternative cell death pathways which may function as back up cell death programme for apoptosis. Addition of Z-VAD-fmk may block apoptotic cell death while sensitizing cells to necrotic or autophagic cell death [23].

### PARP degradation is associated with FeNG induced apoptosis

Activation of caspase 3 subsequently leads to apoptotic cell death through cleavage of broad spectrum of cellular target protein including poly (ADP-ribose) polymerase (PARP). In many cellular systems undergoing apoptosis, the endogenous PARP 116 kDa protein is cleaved to generate an 89 kDa fragment [24]. Therefore we investigated the change of PARP status in CEM/ADR5000 cell after FeNG treatment by western blot analysis. As shown in the fig. 7C and 7D cleaved 89 kDa fragments were appeared after FeNG treatment in a time dependent manner.

## Discussion

Cross-resistance to multiple classes of chemotherapeutic agents is a major problem in the treatment of several types of human cancers [25], [26]. A major mechanism of this resistance is the enhanced efflux of a wide variety of structurally distinct classes of chemotherapeutic agents due to the overexpression of P-gp [27].

In this present communication we report that the synthesis and biological characterization of novel iron complex (FeNG), which have cogent *in vitro* anti proliferative and cytotoxic potential against drug resistance T lymphoblastic leukemia (CEM/ADR5000) cell line.

The primary objective of this study was to develop a water soluble, non-toxic metal complex, which have anticancer potential. We also attempted to determine whether this complex could selectively kill drug resistance cancer cells, leaving non-malignant cells viable. In addition, we also dissected the underlying key molecular events associated with iron complex mediated anti proliferative effect in CEM/ADR5000 cell line.

Initial cytotoxicity studies were carried out to determine the IC_50_ values of FeNG in different human cell types. The use of three different cell types allows us to determine whether FeNG has differential effects on drug resistant and drug sensitive T lymphobastic leukemia cell lines. Moreover, through the use of human PBMC as non-malignant normal cell, may provide a means by which the potential selective nature of the complex can be identified. The result obtained from initial cytotoxicity studies showed that following 24 h to 72 h exposure the complex displayed both the concentration and time dependent anti proliferative effects on CEM/ADR5000 and CCRF-CEM cell line. On the contrary the complex kept the non-malignant normal PBMC viable during exposure within stipulated experimental concentration and time frame. We have also tested the cytotoxic activity of metal free ligand and simple aquated iron metal ion on three different cell types but none of cells show significant toxicity (Data not shown) towards both of the components. Based on IC_50_ values presented in table 1, it was found that both drug resistant and sensitive cells were more or less equally susceptible to FeNG induced cytotoxicity. In addition the results presented in table 2 show that the resistance factor (RF) for doxorubicin is significantly higher compared to iron complex suggesting that the complex is not a potential MDR1 substrate.

The morphology of the drug treated cells is used to determine the extent and nature of cytological effects. Hoechst 33342 stain is used to determine nuclear morphology and DNA condensation result presented in the fig. 3A and 3B clearly shows that FeNG induces nuclear fragmentation and DNA condensation in a time dependent manner in CEM/ADR5000 cell line. This result indicates a clue that apoptosis may be involved in FeNG induced cell death in CEM/ADR5000 cell line as the nuclear fragmentation and DNA condensation are hallmark for apoptosis. To substantiate this notion, we carried out annexin V/PI binding assay; we have found that FeNG increases the percentage of annexin V positive population in time dependent manner whereas the percentage of both annexin V and PI positive population remains negligible as time progressed. These results clearly indicate that FeNG kills CEM/ADR5000 cells through induction of apoptosis. Further studies on cell cycle analysis of CEM/ADR5000 cells after FeNG treatment reveals an increase in the sub diploidal population which represent cells with significant DNA damage indicating a late apoptotic stage with respect to cycling cells. However, FeNG does not affect cell cycle check points in CEM/ADR5000 cell line within experimental time frame.

Apoptosis follows two main pathways, the extrinsic pathways, initiated by binding of ligand of specific death receptor and the intrinsic pathways initiated at mitochondria. To draw an inference about pathways involves in FeNG induced apoptosis, FasR expression has been checked and we have found no FasR expression on CEM/ADR5000 cells FeNG post treatment. This apparently suggests that extrinsic pathway may not be involved in FeNG induced apoptosis. As regards the mitochondrial pathway, the most critical events during apoptosis are the release of cytochrome c from mitochondria into cytosol, after development of the mitochondrial transition pore. Cytochrome c in the cytoplasm complexes oligomerizes apoptosis activating factor 1, leading to activation of Caspase 9 and the effector caspase cascade. The translocation of cytochrome c to cytoplasm generally occurs simultaneously with the decrease of ΔΨ_m_, another marker of subsequent cell death. The release of cytochrome c and the change in ΔΨ_m_ are the key events in intrinsic pathway of apoptosis. Indeed, we investigated the effect of FeNG on mitochondrial membrane potential and found that initially FeNG increases the ΔΨ_m_ up to 6 h and then ΔΨ_m_ decreases steadily in time dependent fashion and translocation of cytochrome c into cytoplasm also occurrs in time dependent manner after FeNG treatment. This early hyper polarization event, described by other authors for different cell types, seems to represent a prerequisite for rapid mitochondria mediated apoptotic cell death that eventually leads to the loss of ΔΨ_m_ [28], [29], [30]. Our data discloses that mitochondrial apoptosis pathway may be involved in FeNG mediated apoptosis. In the present study, we have demonstrated that FeNG induces generation of ROS and mitochondrial dysfunction but NAC blocks the ROS production and abrogates FeNG induced apoptosis in CEM/ADR5000 cells. Furthermore, FeNG also deplete intracellular GSH. This result indicates that the generation of ROS and intracellular GSH depletion i.e. cellular redox imbalance may play an important role in FeNG induced apoptosis. At present it is not perceptible how FeNG induces production of ROS and disruption of mitochondrial function. However, one possible explanation is that FeNG directly or indirectly interacts with the ROS generating system resulting in an increase in the production of O_2_^-^, or increased amount of H_2_O_2_ produced by FeNG, later may lead to the formation of highly damaging hydroxyl radical by Fenton reaction.

Evidence suggests that most proapoptotic stimuli induce activation of a family of intracellular cystein protease called caspases. Activation of Caspase 3 followed by PARP cleavage in CEM/ADR5000 cells occurred after exposure to FeNG which represents the irreversible or execution stage of apoptosis. Although caspase 3 activity is associated with FeNG induced apoptosis, but Caspase 3 specific inhibitor Ac-DEVD-cho didn't completely protect CEM/ADR5000 cell from FeNG induced apoptosis. On the other hand ROS scavenger NAC completely abrogate FeNG induced apoptosis (fig. 4B) and caspase 3 activation (data not shown).

In summary, our data provide evidence that novel non-toxic iron complex selectively kills cancer cells through induction of apoptosis in mitochondrial pathway in CEM/ADR5000 cells and reactive oxygen species play a pivotal role in iron complex mediated apoptosis. In conclusion the present report suggests that FeNG is a potent *in vitro* growth suppressing agent for T lymphoblastic leukaemia cell line irrespective of their multi drug resistance status and may have tremendous therapeutic potential as anti leukemic drug.

## Materials and Methods

### Reagents

N-(2-hydroxy) acetophenone, glysine, ferrous sulphate, MTT dye (3-[4, 5-dimethylthiazol- 2-yl]-2, 5-diphenyltetrazolium bromide, N acetyl cystein (NAC) were purchased form Sigma Chemical Chompany,St. Louis, MO, propidiam iodide, 2′, 7′-dihydrodichlorofluorescin diacetate (H2-DCFDA, Molecular Probes), FITC-labeled Annexin V, o-phthalaldehyde, 5,5′,6,6′-tetrachloro-1,1′,3,3′- tetraethylbenzinidazolylcarbocyanine iodide JC-1 dye (Molecular ProbesTM, Invitrogen), anti-PARP antibody (Santacruz), anti FasR antibody (Santacruz), anti-cytochrome *c* antibody (BD PharMingen, San Diego, CA), HRP-conjugated secondary antibody (Sigma Chemical Chompany,St. Louis, MO).

### Synthesis of the ligand

The ligand, PHAG was prepared according to the reported methods [7]. In brief, a cold aqueous solution of KOH (1.03 g, in 12 ml) was mixed with cold aqueous solution of glycine (1.38 g in 12 ml) and held at 15–20°C in an ice bath with continuous stirring. An ethanolic solution of 2-(Hydroxy) acetophenone (2.5 g in 25 ml) was added drop wise. Deep yellow color was developed and stirring was continued for 1 h followed by 5 h at room temperature. The solvent was removed by a rotary evaporator. The yellow mass was washed with pet-ether and precipitated with methanol-diethyl ether mixture. The crude product was recrystallised from methanol to yield PHAG. Yield 75%, m.p.258°–260°C.

### Synthesis of the iron complex

Ferrous N-(2-hydroxy acetophenone) glycinate (FeNG) was synthesized from the ligand, potassium N-(2-hydroxy acetophenone) glycinate by its reaction with ferrous sulphate; in brief, 460 mg potassium (N-2-hydroxyacetophenone) glycinate (NG) and 280 mg ferrous sulphate was dissolved in 5 ml double distilled water separately. Both the solutions were cooled to 8–10°C. The solution of PHAG was added dropwise to ferrous sulphate solution kept in ice bath. The mixture was rotated in a magnetic stirrer for 25–30 mins. maintaining the temperature at 7–8°C. deep brown precipitate deposited and was allowed to settle for 30 mins. in refrigerator. The precipitate was isolated by centrifugation and recrystallised in water-alcohol. Yields 40%, mp.>400°C, *Anal*. Calc. C_10_H_13_O_5_NFe: C, 39.6, H, 3.3, N, 4.62; Found: C, 38.25; H, 3.28; N, 4.72.

### Chemical characterization

UV-vis spectra was recorded in Shimadzu UV 160 A and in Varian Cary 100 Scan in the range of 800−200 nm.

IR spectra were recorded in Perkin-Elmer RX 1 FT spectrophotometer in KBR discs in the range 4500−500 cm^−1^.

Proton NMR spectra was recorded in DMSO-d6 on a Bruker ACF 300 spectrometer at 300.13 MHz reference to Me_4_Si (0.0 ppm).

Mass spectrum was recorded in an AEI MS-30 mass spectrometer.

C, H, N was measured by Perkin-Elmer 2400 Series II CHN analyzer.

### Cell culture

The human T-cell acute lymphoblastic CCRF-CEM and CEM/ADR5000 leukemia cell lines [5], [13] were maintained in RPMI medium (GIBCO Invitrogen Corp., Carlsbad, California, USA) supplemented with 5% fetal bovine serum (FBS), additional glutamine (0.15%), HEPES (25 mM) and 50 mg/ml gentamycin. Cells were grown in plastic tissue culture flasks (Greiner Bio-One, Germany) in a 5% CO_2_ atmosphere at 37°C. Cells were passages twice weekly. The doxorubicin resistant CEM/ADR5000 cell line was generated by treating CCRF-CEM cells with doxorubicin doses up to a final concentration of 5000 ng/ml doxorubicin [31].These cell lines were kindly provided by Prof T Efferth, University of Mainz, Germany. The CEM/ADR5000 specifically overexpress P glycoprotein without concomitant over-expression of MRP1 or BCRP [32], [33]. Furthermore, the cross-resistance profile of CEM/ADR5000 cells to a broad range of established anti-cancer drugs and investigative novel compounds have been analyzed [34]. Cells from exponentially growing cultures were used for all experiments. All experiments were repeated three times.

### Isolation of PBMC

Heparinized peripheral blood of human was taken and diluted with equal volume of RPMI 1640. Lymphocyte-enriched mononuclear cells were isolated by Histopaque 1077 (Sigma) density gradient centrifugation of diluted blood was washed, and finally resuspended in cold RPMI 1640 supplemented with 5% heat inactivated fetal bovine serum (RPMI-FBS).

### Treatment

A 10^−2^ M solution of FeNG was prepared just before the experiments by dissolving the lyophilized compounds in water. For MTT assay treatments were performed with a concentration of 10^−3^ M to 10^−8^ at 37°C in medium supplemented with serum. As control, equal volumes of medium were added to untreated cells. The pancaspase inhibitor zVAD-fmk, caspase 3 specific inhibitor Ac-DEVD-cho (BD bioscience) and antioxidant N acetyl cystein (NAC) was used at a final concentration of 20 µM, 50 µM and 2.5 mM to 10 mM respectively, preincubated for 1 h before the addition of FeNG, and maintained throughout the experimental time. 10^−4^ M concentration of FeNG (above IC_50_ value for CEM-ADR cell) was used for subsequent experiment unless otherwise specified.

### Cytotoxicity assay (MTT assay)

The data generated were from three separate experiments, each performed in duplicate. Cell viability was determined using the MTT assay, which was carried out as described previously [35] with slight modification, briefly, cells were seeded in 96-well plates at a density 4×10^4^ of cells per well. For single-agent studies, cells were seeded and allowed to settle for 24 h before treatment with increasing concentrations of drug and incubate it for further 72 h with 5% CO_2_ at 37°C. After completion of incubation cells were incubated with 0.4 mg per ml of MTT dye (3-[4, 5-dimethylthiazol- 2-yl]-2, 5-diphenyltetrazolium bromide; Sigma, France) for 4 h at 37°C. The monolayer was suspended in 0.1 ml of DMSO and the absorbance at 560 nm was read by ELISA reader (Tecan 200). The control value corresponding to untreated cells was taken as 100% and the viability of treated samples were expressed as a percentage of the control. The IC_50_ values were determined as the concentration that reduced cell viability by 50%. The Resistance Factor (RF) was calculated by dividing the drug toxicity (IC_50_ value) observed in the multi-drug resistant positive cells by the drug toxicity in the multi-drug resistant negative cells.

### Determination of Nuclear fragmentation

Morphological determination of levels of apoptosis was performed by labelling the cells with the nuclear stain Hoechst 33258 and visualisation by fluorescence microscopy. Briefly, CEM ADR cells were treated with FeNG for various periods. Cells were washed with ice-cold PBS and fixed with 1% Para formaldehyde. The suspensions were then washed with PBS and stained with Hoechst 33258 (5 µg/ml). Nuclei (blue) that were condensed or fragmented were scored as apoptotic.

### Cell cycle analysis

Cell cycle analysis was studied by flow cytometry. In brief, cells were seeded in 90 mm tissue culture plate and treated with drugs. At various time points, cells were recovered, washed twice in PBS, fixed in 70% ethanol, and stored at 4°C until analysed. Cells were washed twice in PBS, incubated for 1 h at room temperature with 250 µg/ml RNAse A and 20 min at 4°C with 20 µg/ml propidium iodide (PI). The cell cycle distribution and percentage of apoptotic cells were determined using a FACS calibur flow cytometer (Becton Dickinson, USA). Ten thousand events were analysed for each sample. Appropriate gating was used to select the single-cell population. The same gate was used on all samples, ensuring that the measurements were made on a standardised cell population.

### Annexin V binding assay

Staining the cells with Annexin V-FITC and propidium iodide (PI) can be used in a bivariate analysis to distinguish between cells undergoing apoptosis (PI negative) and those that are necrotic or dead (PI positive). Cells (2×10^5^) were incubated with FITC-labeled Annexin V and propidium iodide (PI) at room temperature for 15 min in the dark and analyzed using a FACS Calibur (Becktone Dickinson).

### Determination of FasR expression by Facs analysis

To quantitate CD95 or FasR expression of CEM ADR cell after FeNG treatment, or left untreated and human recombinant IFN-γ treatment (positive control) were rinse with PBS twice and were incubated with anti FasR primary antibody at room temperature (RT) for 45 mins. After that cells were washed thrice with PBS with 3% FBS an incubated with FITC conjugated secondary antibody at RT for 30 mins. Negative controls were incubated with secondary antibodies only. Final volume was adjusted to 500 ml with PBS, and labeling was analyzed by flow cytometry by using a FACS or fluorescence-activated cell sorter and CELLQuest software (BD Biosciences, San Jose, CA). A minimum of 10^4^ cells was counted for each sample. The gate was set to exclude approximately 99.5% of the negative control cells. At least duplicate independent measurements of the effects of each treatment were performed.

### Intra cellular ROS accumulation study

Levels of ROS generation in cells were assessed fluorometrically using 2′, 7′-dihydrodichlorofluorescin diacetate (H2-DCFDA, Molecular Probes). H2-DCFDA is a nonfluorescent, cell-permeant compound. Endogenous esterases within the cell cleave the acetate groups, thus trapping the reduced form of the probe (DCHF) intracellularly. It is known that the probe can be readily oxidized to DCF by H_2_O_2_ or OH. Cells were treated with drug or left untreated for 1 to 6 h and 8 h. The cells were then washed with PBS and further incubated with H2-DCFDA for 30 min at 37°C in dark. After incubation, cells were washed twice in PBS at room temperature for 5 min each time. The fluorescence was measured at excitation and emission wavelengths of the oxidized form were 488 nm and 525 nm respectively [36].

### Determination of intracellular GSH contents

Determination of cellular GSH content was performed by a modification of the method of [37]. Drug treated and untreated cells were washed twice with PBS and cell pellet (10^6^ cells) was resuspended in 0.5 ml ice cold distilled water and 0.2 ml of a solution containing 17.5% HPO3 was added to it. After centrifugation (10 min, 3000 rpm) 0.25 ml of the supernatant was mixed with 0.25 ml of 0.1 M-sodium phosphate buffer containing 5 mM-EDTA (pH 8.0), and 300 µl of the mixture was added to 1.6 ml of the phosphate/EDTA buffer (pH 8.0) and 100 µl of o-phthalaldehyde (0.1% in methanol). After 15 min at room temperature the fluorescent GSH adduct was determined (excitation, 350 nm; emission, 420 nm) by fluorescence spectrophotometer.

### Determination of mitochondrial membrane potential

The lipophilic cationic probe 5,5′,6,6′-tetrachloro-1,1′,3,3′- tetraethylbenzinidazolylcarbocyanine iodide JC-1 dye (Molecular ProbesTM, Invitrogen) was used to measure mitochondrial inner membrane potential (ΔΨ_m_) in drug treated or untreated cells (5×10^5^) grown in 35 mm tissue plates. JC-1 accumulates in the mitochondria in proportion to ΔΨ_m_, forming aggregates that fluoresce red. In the cytoplasm, JC-1 exists as monomers that fluoresce green. The ratio of red-to-green fluorescence is proportional to ΔΨ_m_. Red fluorescence (excitation, 570 nm; emission, 595 nm) and green fluorescence (excitation, 485 nm; emission, 535 nm) were measured using a Varion spectrofluorimeter following 30 mins incubation with 5 µM JC-1 at dark in a 5% CO_2_ atmosphere at 37°C incubator [38].

### Caspase 3 activation assay

Cells washed in PBS and resuspended in 25 mM Hepes (pH 7.5), 5 mM MgCl2, 5 mM EDTA, 5 mM dithiothreitol (DTT), 2 mM phenylmethylsulfonyl fluoride (PMSF), 10 mg/ml pepstatin A, and 10 mg/ml leupeptin after treatment. The kit (Caspase fluorometric assay system) was used to investigate caspase activity. Cells were lysed and clarified using centrifugation at 1,2000 *g* for 5 min. The clear lysates containing 50 mg of protein were incubated with 50 mM substrate Ac-DEVD-AMC at 30°C for 1 h. Levels of released AMC were measured using a spectrofluorometer (Varion) with excitation at 360 nm and emission at 460 nm (Caspase Assay System, BD bioscience, USA).

### Western blot analysis

Following FeNG treatments, cells were washed twice with ice-cold PBS. Cell pellet was resuspended in 100 µl of cell lysis buffer containing 20 mM Tris–HCl, pH 7.4, 150 mM NaCl, 1% Triton X-100, 1 mM EDTA, 1 mM EGTA, 0.5 mM phenyl methyl sulfonyl fluoride, 1 mM sodium orthovanadate, 0.5% NP-40, 5 U/ml aprotinin and protease inhibitor cocktail. After 30 min incubation on ice cell lysate was cleared by centrifugation at 12000 rpm for 15 min at 4°C. Protein concentration in lysates was determined by Bradford method. For immunoblot analyses, 100 µg of protein lysates per sample were denatured in 2× SDS–PAGE sample buffer and subjected to SDS– PAGE on 10% Tris–glycine gel. The separated proteins were transferred onto PVDF membrane followed by blocking with 5% BSA (w/v) in TBS (10 mM Tris, 100 mM NaCl, 0.1% Tween 20) for 1 h at room temperature. Membrane was probed with anti-PARP antibody (Santacruz) overnight at 4°C followed by 1 h incubation with HRP-conjugated secondary antibody and using a chemiluminescence kit (Lumi Glow, Cell signalling technology).

### Preparation of cytosolic extract and immunoblot analysis of cytochrome c release

To carry out cytochrome c translocation studies, cellular sub fractionation was performed as previously reported [39] with minor modifications. At the end of FeNG treatment, cells were washed twice with ice-cold PBS. The cell pellet was resuspended in 300 µl of extraction buffer containing 200 mM mannitol, 70 mM sucrose, 20 mM HEPES–KOH, pH 7.4, 50 mM KCl, 5 mM EGTA, 2 mM MgCl_2_, 0.1 mM PMSF and protease inhibitors (Complete Cocktail; Marck bioscience). After 20 min incubation on ice, cells were homogenized by 30–40 strokes with a glass Dounce homogenizer on ice, and resulting homogenates were left on ice for an additional 20 min. Homogenates were centrifuged at 600 *g* for 15 min at 4°C, and resulting supernatant was further centrifuged at 14 000 *g* for 30 min at 4°C, to yield cytosolic extract. 70 µg protein per sample was resolved on 15% SDS–PAGE and transferred onto PVDF membrane followed by blocking in 5% (w/v) bovine serum albumin (BSA) in TBS. Membrane was probed with anti-cytochrome *c* antibody (BD PharMingen, San Diego, CA) overnight at 4°C followed by 1 h incubation with HRP-conjugated secondary antibody and using a chemiluminescence kit (Lumi Glow, Cell signalling technology).

### Densitometric analysis

Immunoreactive bands of Cytochrome C, PARP1 and β actin were scanned (Bio-Rad, model GS800) and then images were digitized and analyzed by using Bio-Rad QUANTITY 1 software. Immunoreactive bands were quantitated and expressed as the ratio of each band density to corresponding loading control (β actin) band density and values were represented after normalization to untreated control.

### Statistical analysis

All data reported are the arithmetic mean from three independent experiments performed in triplicate ±S.D. unless stated otherwise. The unpaired Student's t-test was used to evaluate the significance differences between groups, accepting P<0.05 as a level of significance. Data analyses were performed using the Prism software (GraphPad, San Diego, CA).
